# Novel Avian Influenza A(H5N6) Virus in Wild Birds, South Korea, 2023

**DOI:** 10.3201/eid3006.240192

**Published:** 2024-06

**Authors:** Andrew Yong Cho, Young-Jae Si, Dong-Ju Kim, Ye-Ram Seo, Dong-Yeop Lee, Daehun Kim, Dongbin Lee, Yaemoon Son, Hyesung Jeong, Chang-Seon Song, Dong-Hun Lee

**Affiliations:** Wildlife Health Laboratory, Seoul, South Korea (A.Y. Cho, Y.-R. Seo, D.-Y. Lee, D.-H. Lee);; Avian Disease Laboratory, Seoul (A.Y. Cho, Y.-R. Seo, C.-S. Song);; National Institute of Wildlife Disease Control and Prevention, Gwangju, South Korea (Y.-J. Si, D.-J. Kim, D. Kim, D. Lee, Y. Son, H. Jeong);; Konkuk University Zoonotic Disease Research Center, Seoul (C.-S. Song, D.-H. Lee)

**Keywords:** avian influenza virus, highly pathogenic avian influenza virus, H5N6, clade 2.3.4.4, wild bird, genomic surveillance, influenza, viruses, zoonoses

## Abstract

We isolated novel reassortant avian influenza A(H5N6) viruses containing genes from clade 2.3.4.4b H5N1 virus and low pathogenicity avian influenza viruses in carcasses of whooper swans and bean geese in South Korea during December 2023. Neuraminidase gene was from a clade 2.3.4.4b H5N6 virus infecting poultry and humans in China.

Infection caused by highly pathogenic avian influenza viruses (HPAIVs) have caused major economic losses in the poultry industry and pose a serious threat to public health. The A/goose/Guangdong/1/1996 (gs/GD) lineage of H5 HPAIV emerged in China in 1996 and diverged into 10 genetically independent hemagglutinin (HA) clades (0–9) and subclades (*1*). The gs/GD lineage of H5 HPAIV has caused outbreaks worldwide, infecting a range of wildlife, poultry, and humans (*1*). Clade 2.3.4.4 H5Nx HPAIV containing multiple neuraminidase (NA) subtypes (*2*) has dominated outbreaks worldwide from 2014 onwards and further divided into subclades 2.3.4.4a–h (*3*). Currently, clade 2.3.4.4b H5N1 HPAIV is predominant globally after causing outbreaks in Europe in the fall of 2020 and in Africa, the Americas, Asia, and Antarctica (*4*–*7*).

During October 2022–March 2023, a total of 16 different genotypes of H5N1 2.3.4.4b HPAIV caused outbreaks in South Korea, including 174 cases in wild birds (*8*). Based on the available surveillance data, no new virus incursions have occurred in South Korea during summer and fall 2023. National surveillance for HPAIV began in South Korea in the fall of 2023. We isolated 3 H5N6 HPAIVs from wild bird carcasses found in South Korea during December 2023 (A/whooper swan/Korea/23WC075/2023[H5N6], A/whooper swan/Korea/23WC116/2023[H5N6], and A/bean goose/Korea/23WC111/2023[H5N6]) (Appendix Table 1). We conducted next-generation sequencing of the isolates and shared complete genome sequences publicly. We conducted comparative phylogenetic analysis to infer the origin and evolution of the viruses.

All H5N6 isolates were identified as HPAIVs based on the presence of multiple basic amino acids at the HA proteolytic cleavage site (REKRRKR/GLF). BLAST inquiries of the GISAID database (https://www.gisaid.org) indicated all 8 genes shared the highest nucleotide sequence identity (99.77%–100%) with a clade 2.3.4.4b H5N6 virus identified from a peregrine falcon in 2023 in Japan (A/peregrine falcon/Saga/4112A002/2023[H5N6]), harboring the same genome constellation as the H5N6 viruses in South Korea (Table). The HA gene clustered with the major genotype a of the H5N1 clade 2.3.4.4b HPAIV that circulated during 2022–2023 in South Korea (*8*). The NA gene of H5N6 virus clustered with H5N6 HPAIV from China, previously isolated in poultry and humans in 2018, but other internal genes were genetically distinct. Polymerase basic 1 and matrix protein genes also clustered with 2022–2023 H5N1 HPAIVs from South Korea. Polymerase basic 2, polymerase acidic, nucleoprotein, and nonstructural genes clustered with low-pathogenicity avian influenza viruses in Eurasia (Appendix Figure 2).

**Table:** Nucleotide sequence identities between gene segments of novel clade 2.3.4.4b highly pathogenic avian influenza A(H5N6) virus isolate A/whooper swan/Korea/23WC075/2023 from South Korea and nearest top 3 homologs in the GISAID EpiFlu database*

Gene	Top 3 query	Accession no.	Nucleotide identity	% Identity
PB2	A/peregrine falcon/Saga/4112A002/2023 (A/H5N6) segment 1 (PB2)	EPI2898974	2280/2280	100.00
	A/environment/chongqing/1795/2023 (A/H9N2) segment 1 (PB2)	EPI2841012	2271/2280	99.61
	A/environment/Kagoshima/KU-I6/2021 (H4N6) (A/H4N6) segment 1 (PB2)	EPI2553141	2253/2280	98.82
PB1	A/peregrine falcon/Saga/4112A002/2023 (A/H5N6) segment 2 (PB1)	EPI2898975	2273/2274	99.96
	A/egret/Korea/22WC394/2023 (A/H5N1) segment 2 (PB1)	EPI2743089	2271/2274	99.87
	A/common buzzard/Korea/22WC336/2023 (A/H5N1) segment 2 (PB1)	EPI2742993	2271/2274	99.87
PA	A/peregrine falcon/Saga/4112A002/2023 (A/H5N6) segment 3 (PA)	EPI2898976	2150/2151	99.95
	A/common teal/Amur region/92b/2020 (A/H6N2) segment 3 (PA)	EPI1849993	2142/2151	99.58
	A/mallard/Russia Primorje/94T/2020 (A/H1N1) segment 3 (PA)	EPI1849961	2141/2151	99.54
HA	A/peregrine falcon/Saga/4112A002/2023 (A/H5N6) segment 4 (HA)	EPI2898977	1700/1704	99.77
	A/environment/Kagoshima/KU-G4/2022 (H5N1) (A/H5N1) segment 4 (HA)	EPI2789597	1697/1704	99.59
	A/environment/Kagoshima/KU-D4/2022 (H5N1) (A/H5N1) segment 4 (HA)	EPI2789589	1697/1704	99.59
NP	A/peregrine falcon/Saga/4112A002/2023 (A/H5N6) segment 5 (NP)	EPI2898978	1496/1497	99.93
	A/gadwall/Novosibirsk region/3407k/2020 (A/H4N6) segment 5 (NP)	EPI1849870	1479/1497	98.80
	A/mallard/Novosibirsk region/3286k/2020 (A/H4N6) segment 5 (NP)	EPI1849878	1478/1497	98.73
NA	A/peregrine falcon/Saga/4112A002/2023 (A/H5N6) segment 6 (NA)	EPI2898979	1377/1380	99.78
	A/duck/Hunan/S40199/2021(H5N6) (A/H5N6) segment 6 (NA)	EPI1997201	1360/1380	98.55
	A/Changsha/1/2022 (A/H5N6) segment 6 (NA)	EPI2287050	1359/1380	98.48
M	A/peregrine falcon/Saga/4112A002/2023 (A/H5N6) segment 7 (MP)	EPI2898980	982/982	100.00
	A/northern pintail/Kagoshima/KU-64/2022 (H5N1) (A/H5N1) segment 7 (MP)	EPI2794001	980/982	99.80
	A/environment/Kagoshima/KU-B4/2022 (H5N1) (A/H5N1) segment 7 (MP)	EPI2789385	980/982	99.80
NS	A/peregrine falcon/Saga/4112A002/2023 (A/H5N6) segment 8 (NS)	EPI2898981	837/838	99.88
	A/bean goose/Korea/KNU-10/2022 (A/H10N7) segment 8 (NS)	EPI2873490	836/838	99.76
	A/bean goose/Korea/KNU-14/2022 (A/H6N1) segment 8 (NS)	EPI2873460	836/838	99.76
*GISAID, https://www.gisaid.org; HA, hemagglutinin; P, matrix; NA, neuraminidase; NP, nucleoprotein; NS, nonstructural; PA, polymerase acidic; PB, polymerase basic.				

Estimated time to most recent common ancestor (tMRCA) of each gene of the H5N6 viruses and the A/peregrine falcon/Saga/4112A002/2023(H5N6) ranged from February through November 2023 (Figure; Appendix Table 2). On the basis of overlap between the 95% highest posterior density intervals of tMRCA, we assume the novel reassortant H5N6 viruses emerged around August–October 2023 and were introduced into Japan and South Korea. Maximum clade credibility tree of the NA gene revealed the wild bird H5N6 viruses from Japan and South Korea shared a common ancestor with the human infection case of H5N6 virus (A/Changsha/1/2022) from China. Since 2014, a total of 90 human cases of H5N6 infection have been reported in China;w most infections were reported after 2021 (*9*; https://iris.who.int/handle/10665/375483). The tMRCA of the wild bird H5N6 viruses from Japan and Korea and the human infection H5N6 virus from China is estimated to be June 12, 2022 (95% highest posterior density December 7, 2021–November 10, 2022) (Figure; Appendix Table 2). The ancestral H5N6 HPAIV circulating in China potentially donated the NA gene to the clade 2.3.4.4b H5N1 virus in late 2022. The N6 gene of current and ancestral H5N6 HPAIV possessed a stalk deletion potentially acquired during the circulation of viruses in domestic poultry (*10*).

**Figure:**
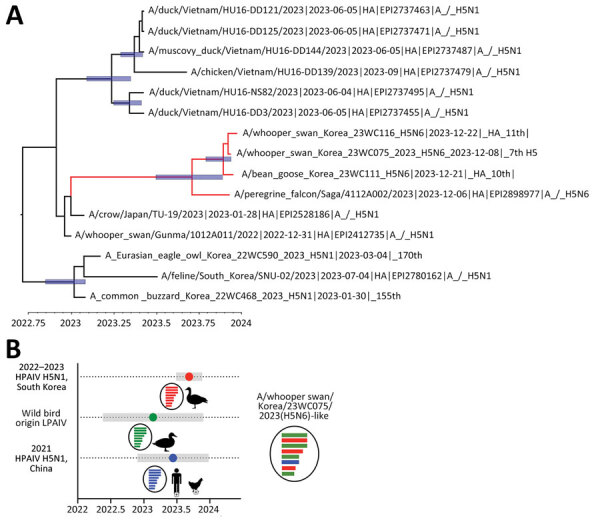
Exploration of most common ancestors for novel reassortant highly pathogenic avian influenza A(H5N6) clade 2.3.4.4 b isolates recovered from wild birds, South Korea. A) Maximum clade credibility tree of viruses found in the carcasses of whooper swans and bean geese in South Korea, December 2023. Tree was constructed using the hemagglutinin gene of the H5N6 viruses. Red indicates H5N6 isolates from South Korea and Japan. The timescale is shown on the horizontal axis in decimal years. Node bars represent 95% highest posterior density of the heights. Accession numbers beginning with EPI indicate isolates from the GISAID database (https://www.gisaid.org). B) Temporal schematic of the reassortant genome constellation of the novel reassortant H5N6 viruses from South Korea. Gene segments originating from H5N1 HPAIVs (red), LPAIVs (green), and H5N6 (blue) HPAIVs are indicated. Shade bars represent 95% highest posterior density range of time to most recent common ancestor. Circles represent the mean time to most recent common ancestor. HPAIV, highly pathogenic avian influenza virus; LPAIV, low-pathogenicity avian influenza viruses.

The H5N6 viruses possessed molecular markers T188, V210, Q222, and G224 in HA, which are associated with binding affinity to α-2,3 sialic acid receptors. We observed S133A and T156A mutations in HA, known to be associated with increased binding to α-2,6 sialic acid receptors. We observed L89V in polymerase basic 2, but not Q591K, E627K, and D701N. We also observed D622G in polymerase basic 1, N30D and I43M in matrix 1, and P42S in nonstructural protein 1, which are associated with increased virulence in mice (*10*).

Previous reports suggest the genomes of clade 2.3.4.4b are evolving through frequent genome reassortments, forming transient and diverse genome constellations change with no apparent pattern of gene segment association (*8*). Detection of H5N6 HPAIVs from wild birds in South Korea and Japan during the 2023–24 wintering season and our phylogenetic analysis suggest H5N6 HPAIVs most likely descended from clade 2.3.4.4b H5N1 viruses circulating during 2022–2023, evolved from reassortment with other low-pathogenicity avian influenza viruses and HPAIVs, and were introduced into South Korea and Japan by wild birds during the fall migration season. Enhanced genomic surveillance of HPAIVs in wild birds is needed for early detection and monitoring of further evolution and spread of viruses.

Appendix Additional information about novel avian influenza A(H5N6) in wild birds, South Korea, 2023.
