# Supplementary material for: Novel Avian Influenza A(H5N6) Virus in Wild Birds, South Korea, 2023
Source: Emerg Infect Dis. 2024 Jun;30(6):1285–8. doi: 10.3201/eid3006.240192 (PMC11139000; doi:10.3201/eid3006.240192)
Supplement: Appendix — Additional information about novel avian influenza A(H5N6) in wild birds, South Korea, 2023. [file 24-0192-Techapp-s1.pdf]

Article DOI: https://doi.org/10.3201/eid3006.240192

EID cannot ensure accessibility for supplementary materials supplied by authors. Readers who have difficulty accessing supplementary content should contact the authors for assistance.

Novel Avian Influenza A(H5N6) in Wild Birds, South Korea, 2023

Appendix

Methods and Materials

Virus isolation

During December 2023, carcasses of two Whooper swan (*Cygnus cygnus*) and one bean goose (*Anser fabalis*) were found in Gumi city of Gyeongsangbuk-do province in South Korea and collected oropharyngeal and cloacal swabs for active HPAIV surveillance in wild birds (Appendix Table 1 and Appendix Figure 1). Swab samples obtained from captured birds were placed in phosphate-buffered saline (PBS) mixed with 0.1% volume of 50 mg/mL gentamicin and thoroughly homogenized by vortexing for 1 min. The supernatant of samples was filtered using a 0.45 µm Minisart Syringe Filter (Sartorius, Göttingen, Germany) and inoculated into 10-day-old specific-pathogen-free (SPF) embryonated chicken eggs. After 72 h of incubation at 37°C, the allantoic fluids were harvested and tested for hemagglutination activity using 10% chicken red blood cells. RNA was extracted from the hemagglutination-activity-positive allantoic fluid using the Maxwell® RSC simply RNA Tissue Kit (Promega, Madison, WI, USA) according to the manufacturer's instruction and screened for the matrix (M) gene and H5 gene of avian influenza virus using real-time reverse transcription-PCR (rRT-PCR) as previously described (*1*).

Genome sequencing and assembly

Complementary DNA was generated using the SuperScript III First-Strand Synthesis system (Invitrogen, Carlsbad, CA, USA) and the eight gene segments were amplified using AccuPrime Pfx DNA Polymerase (Invitrogen, Carlsbad, CA, USA) as previously described (*2*). DNA libraries were prepared using Nextera DNA Flex Library Prep Kit (Illumina, San Diego, CA, USA) with 96 dual-index barcodes according to the manufacturer's instructions. The

complete genome was sequenced using the paired-end 150 Illumina Miseq platform. Trimming and genome assembly of NGS reads were conducted using the QIAGEN CLC Genomics Workbench software.

Phylogenetic analysis

All eight assembled genome sequences were submitted to the BLAST query of the GISAID EpiFlu database (https://gisaid.org/). Top 250 BLAST results were downloaded from the database and removed 100% identical nucleotide sequences using the ElimDupes software (https://www.hiv.lanl.gov/content/sequence/elimdupesv2/elimdupes.html). RAxML v8.0 was used to construct maximum-likelihood tree of each gene using the general time reversible(GTR) model of nucleotide substitution and the Gamma model of among-site rate heterogeneity model with 1,000 bootstrap iterations (*3*). All eight AIV gene sequences were aligned using MAFFT software and used to reconstruct Bayesian relaxed clock phylogeny using BEAST v1.10.4 (*4*,*5*). The GTR + Gamma nucleotide substitution model with an uncorrelated log-normal distribution relaxed-clock method was used, with the Gaussian Markov Random Field (GMRF) Bayesian skyride coalescent prior (*6*). The Markov Chain Monte Carlo (MCMC) of configured xml was run in parallels of five separate chains, each with 100 million steps and samples across chains combined after 10% burn-in. The parameters, each of which had adequate effective sample sizes (>200), were analyzed with TRACER v1.5 (http://tree.bio.ed.ac.uk/software/tracer/) (*7*). A maximum clade credibility (MCC) tree was generated using TreeAnnotator and visualized using FigTree 1.4.4 (http://tree.bio.ed.ac.uk/software/figtree/). The time to most recent common ancestor (tMRCA) was calculated using the height values of the common ancestor node with posterior probability >0.5.

Acknowledgment

We gratefully acknowledge all data contributors, i.e., the Authors and their Originating laboratories responsible for obtaining the specimens, and their Submitting laboratories for generating the genetic sequence and metadata and sharing via the GISAID Initiative, on which this research is based (Technical Appendix 2).

The data support the findings of this study are available in Global Initiative on Sharing All Influenza Data (Technical Appendix 2).

Reference

1. Spackman E, Senne DA, Bulaga LL, Myers TJ, Perdue ML, Garber LP, et al. Development of real-time RT-PCR for the detection of avian influenza virus. Avian Dis. 2003;47(Suppl):1079–82. PubMed https://doi.org/10.1637/0005-2086-47.s3.1079

2. Lee DH. Complete genome sequencing of influenza A viruses using next-generation sequencing. Methods Mol Biol. 2020;2123:69–79. PubMed https://doi.org/10.1007/978-1-0716-0346-8_6

3. Stamatakis A. RAxML version 8: a tool for phylogenetic analysis and post-analysis of large phylogenies. Bioinformatics. 2014;30:1312–3. PubMed https://doi.org/10.1093/bioinformatics/btu033

4. Katoh K, Standley DM. MAFFT multiple sequence alignment software version 7: improvements in performance and usability. Mol Biol Evol. 2013;30:772–80. PubMed https://doi.org/10.1093/molbev/mst010

5. Suchard MA, Lemey P, Baele G, Ayres DL, Drummond AJ, Rambaut A. Bayesian phylogenetic and phylodynamic data integration using BEAST 1.10. Virus Evol. 2018;4:vey016. PubMed https://doi.org/10.1093/ve/vey016

6. Minin VN, Bloomquist EW, Suchard MA. Smooth skyride through a rough skyline: Bayesian coalescent-based inference of population dynamics. Mol Biol Evol. 2008;25:1459–71. PubMed https://doi.org/10.1093/molbev/msn090

7. Rambaut A, Drummond AJ, Xie D, Baele G, Suchard MA. Posterior summarization in Bayesian phylogenetics using Tracer 1.7. Syst Biol. 2018;67:901–4. PubMed https://doi.org/10.1093/sysbio/syy032

Appendix Table 1. Information of HPAIV A(H5N6) from South Korea, Dec 2023

Name	Source	Collection date	Region	GPS coordinates
A/Whooper Swan/Korea/23WC075/2023(H5N6)	Carcass	2023–12–08	Gumi city, South Korea	36°08′22.1″ N, 128°21′9.7″ E
A/Bean goose/Korea/23WC111/2023 (H5N6)	Carcass	2023–12–21	Gumi city, South Korea	36°08′4.3″ N, 128°21′8.3″ E
A/Whooper Swan/Korea/23WC116/2023 (H5N6)	Carcass	2023–12–22	Gumi city, South Korea	36°08′14.9″ N, 128°21′6.3″ E

Appendix Table 2. tMRCA of each gene segment of H5N6 highly pathogenic avian influenza viruses from South Korea, 2023

	tMRCA of H5N6 viruses from Japan and Korea		
Gene	Median	95% HPD range	
PB2	Sep 5, 2023	May 26, 2023	Nov 20, 2023
PB1	October 11, 2023	August 12, 2023	December 1, 2023
PA	July 25, 2023	February 19, 2023	November 26, 2023
HA	September 12, 2023	July 1, 2023	November 20, 2023
NP	May 4, 2023	October 22, 2022	October 21, 2023
NA	June 22, 2023	December 7, 2022	November 21, 2023
MP	November 24, 2023	October 20, 2023	December 6, 2023
NS	February 25, 2023	June 11, 2022	October 2, 2023

HA, hemagglutinin; MP, matrix protein; NA, neuraminidase; NP, nucleoprotein; NS, non-structural; PA, polymerase acidic; PB1, polymerase basic 1; PB2, polymerase basic 2.

.

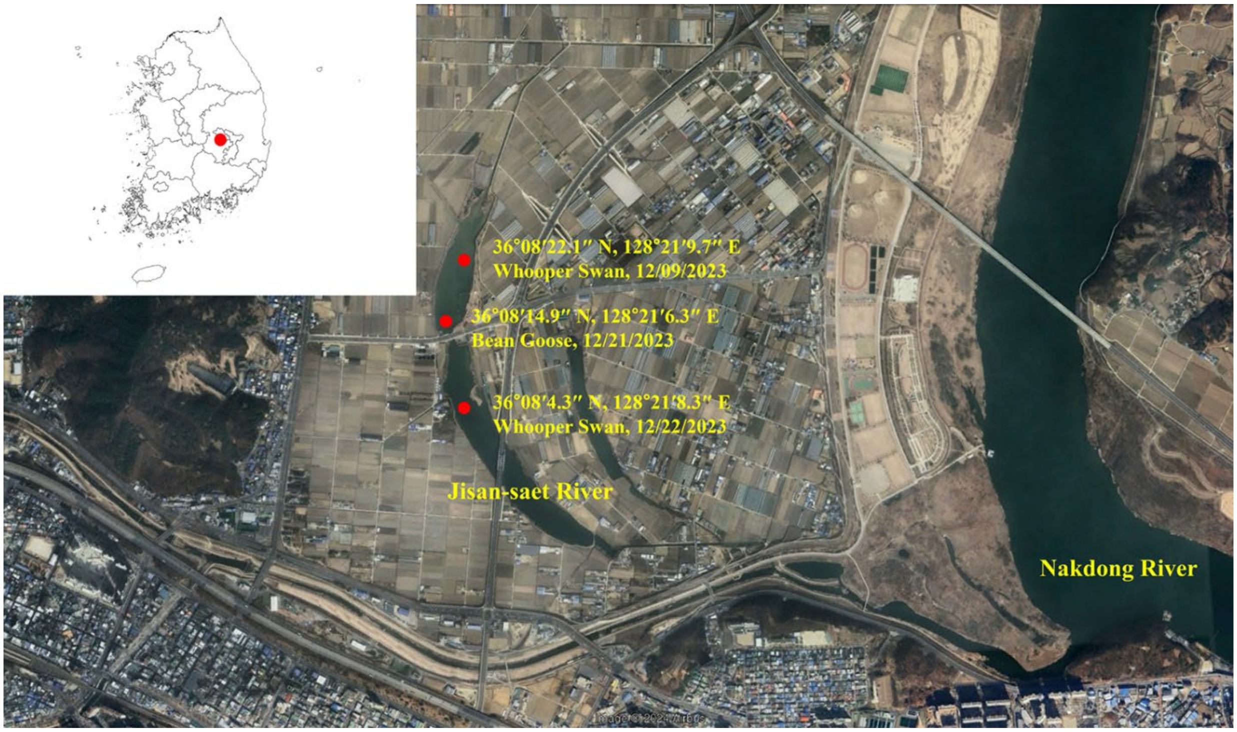

Appendix Figure 1. Location of each carcasses found (indicated with red dots) in Gumi city, South Korea. Map was retrieved from Google Maps (2024 Feb 4, https://www.google.com/maps/@/data = !3m1!1e3!5m1!1e4?entry = ttu).

A

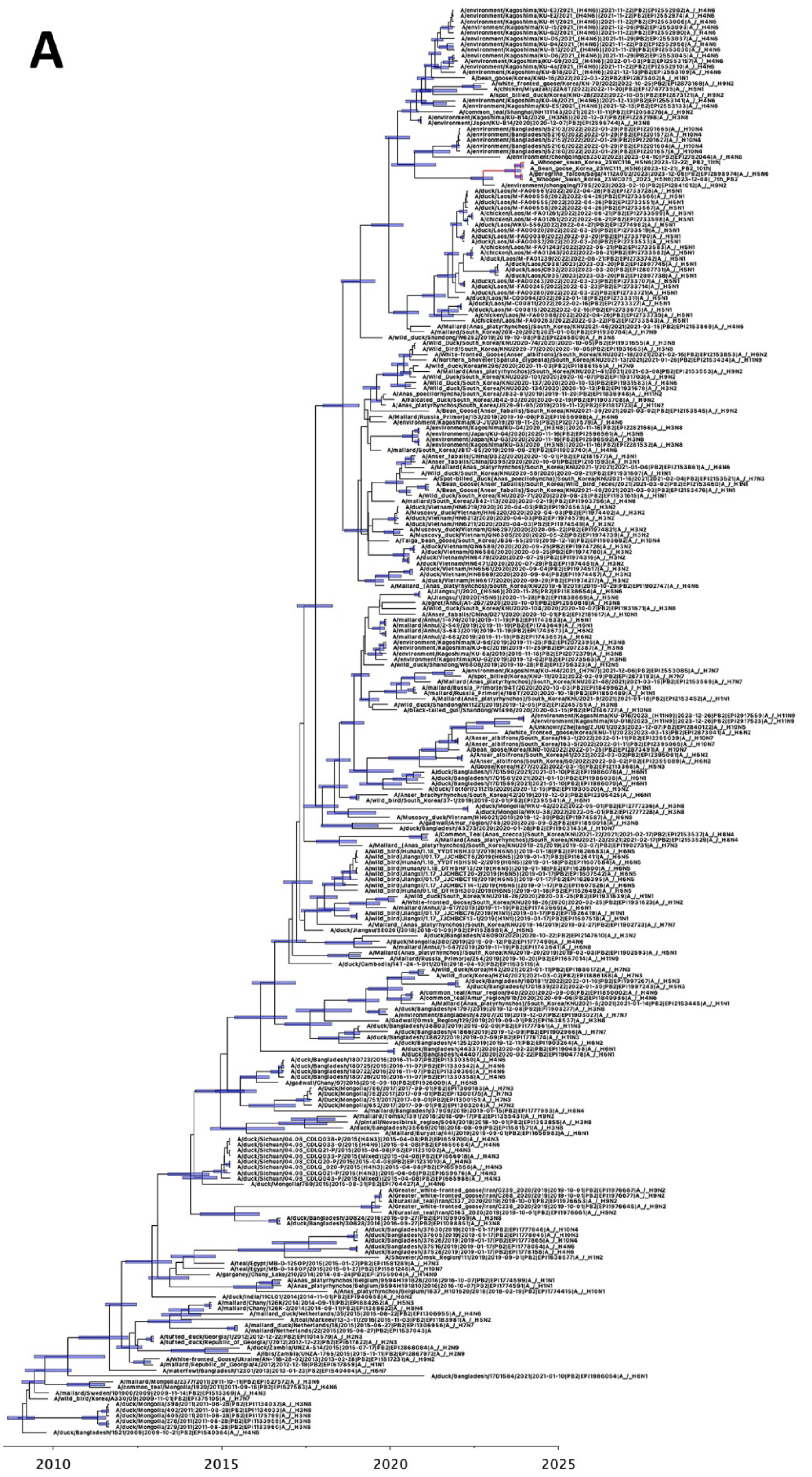

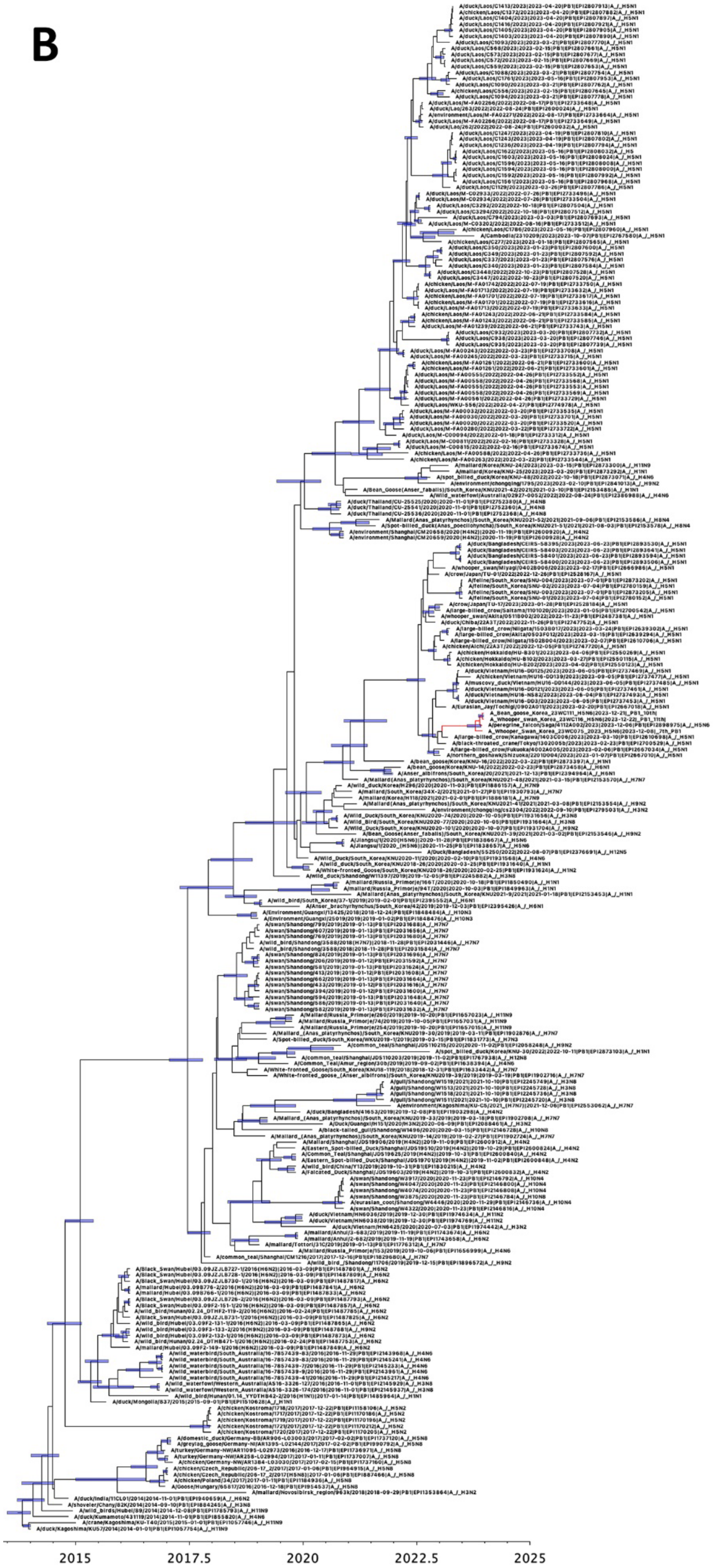
B
2015
2017.5
2020
2022.5
2025

C

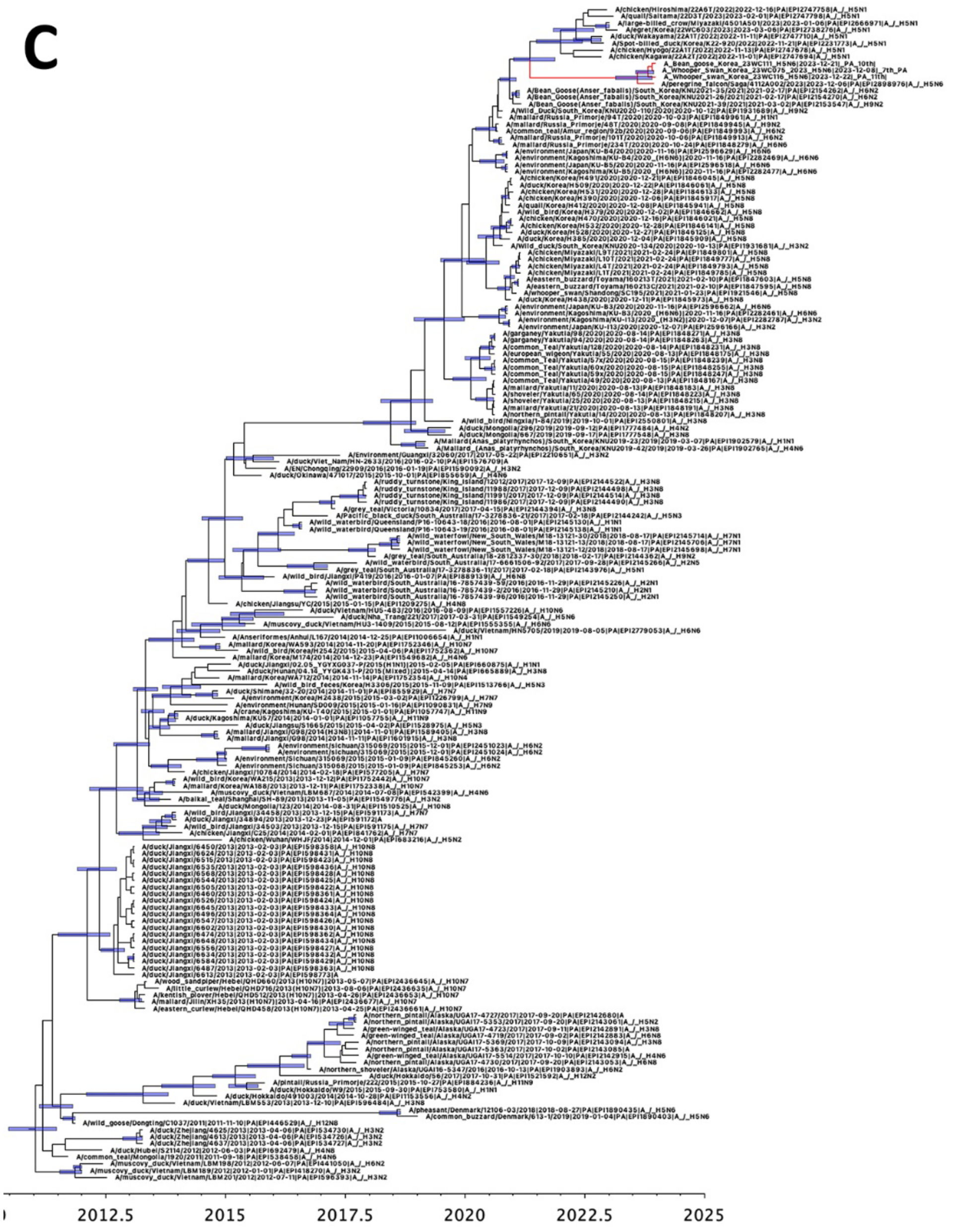

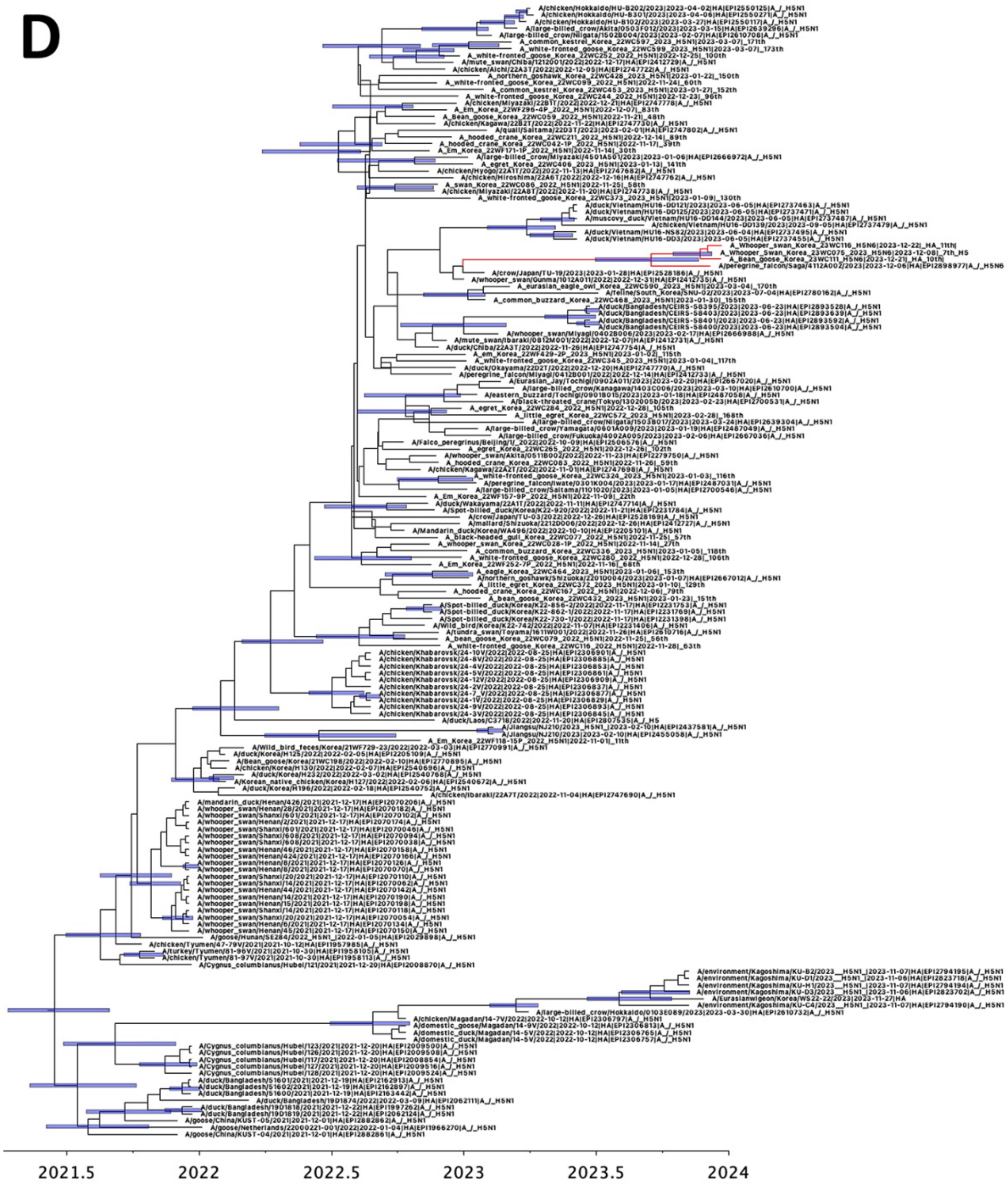
D
2021.5
2022
2022.5
2023
2023.5
2024

E

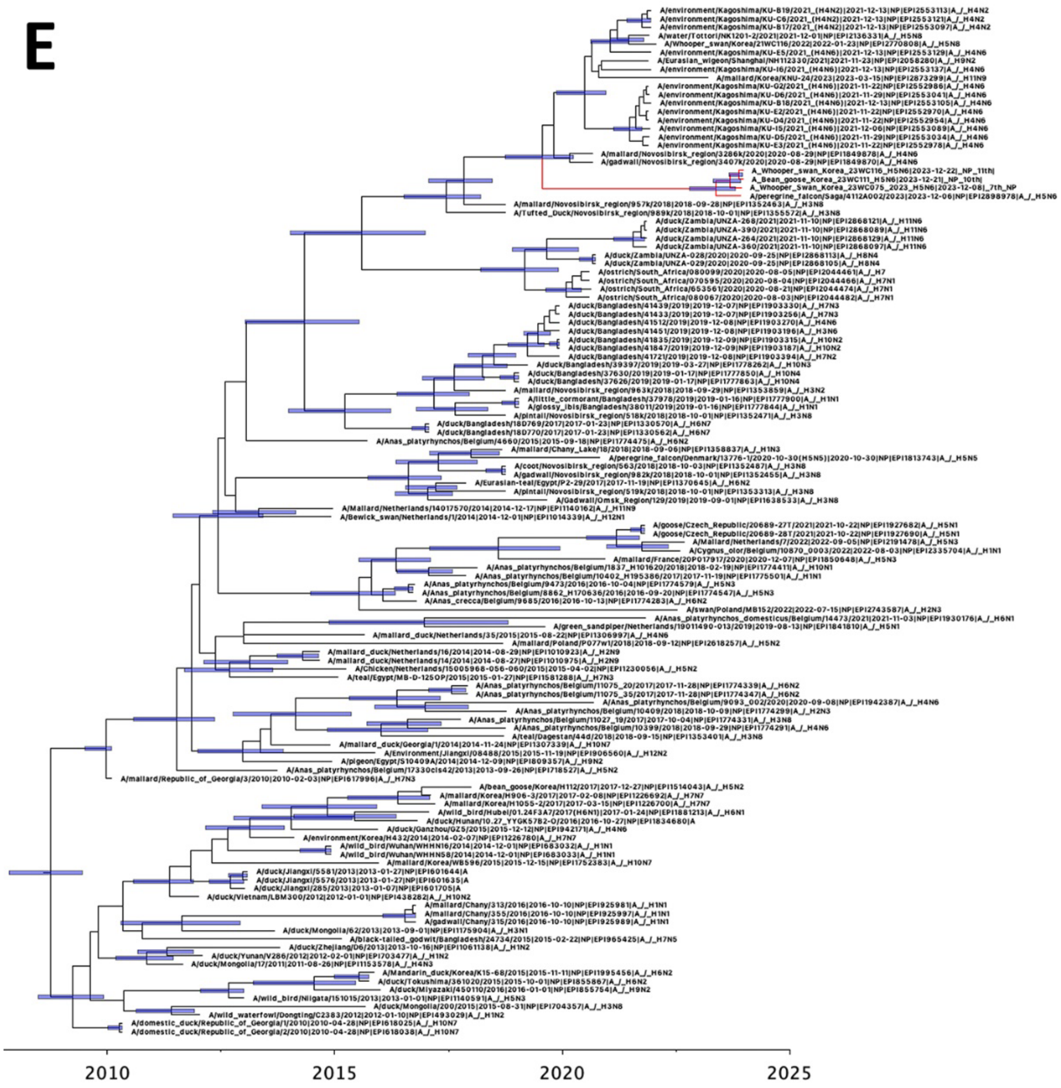

F

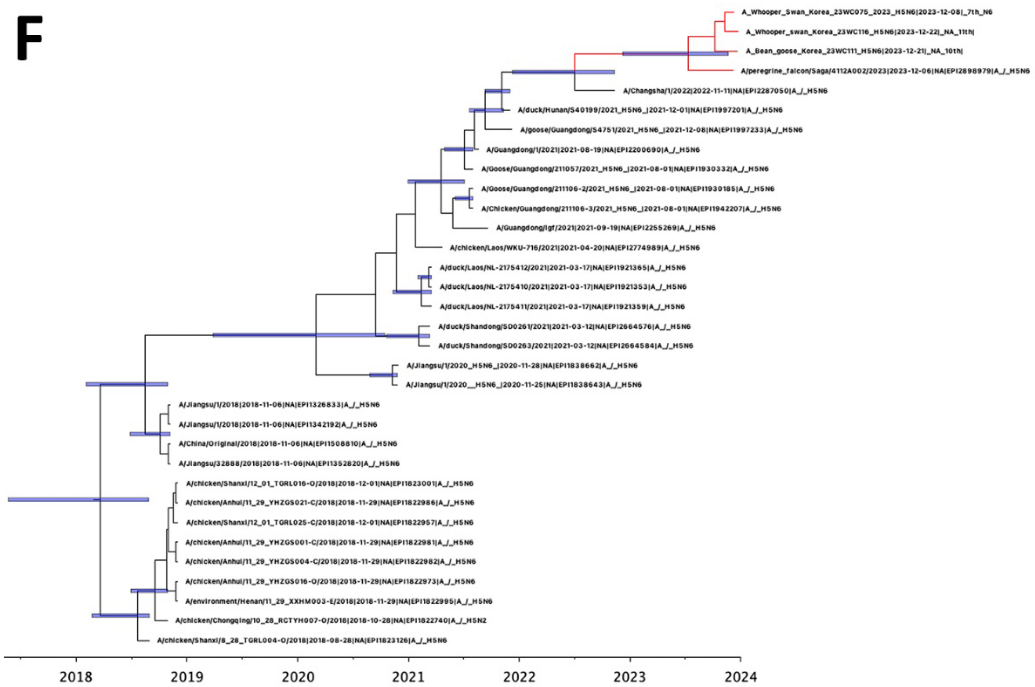
2018
2019
2020
2021
2022
2023
2024

G

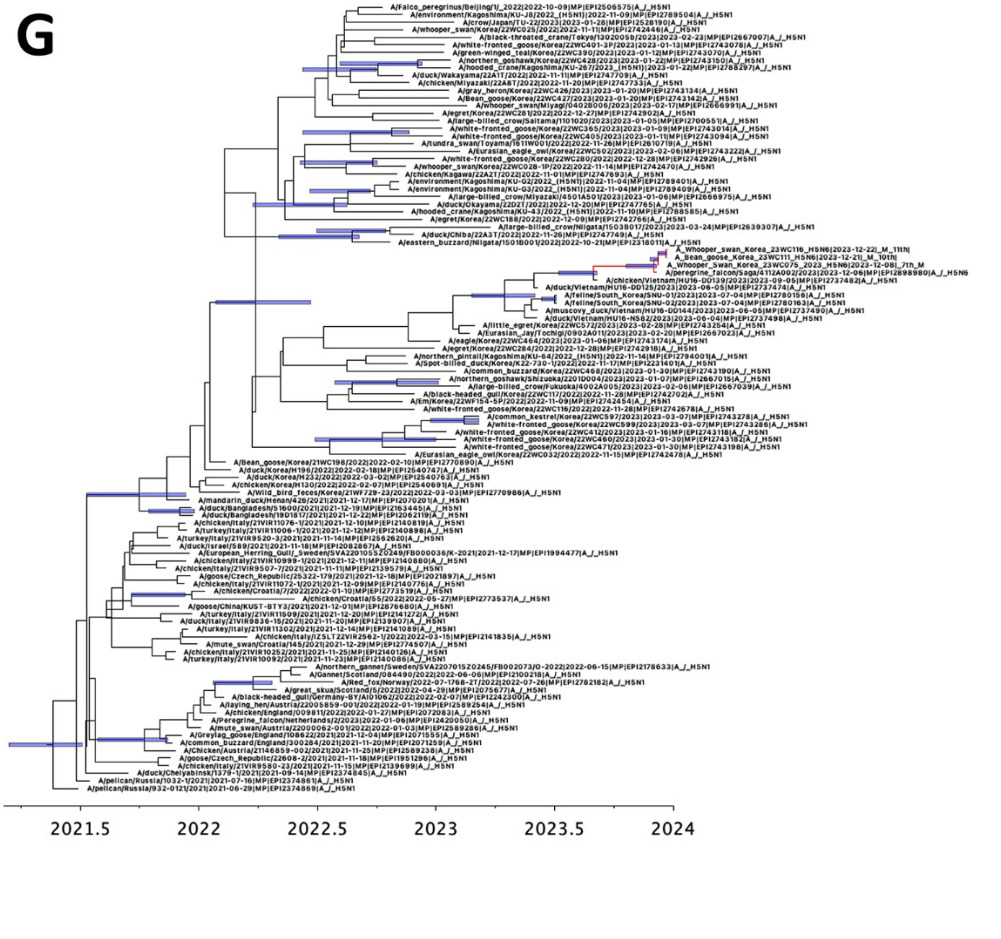
2021.5
2022
2022.5
2023
2023.5
2024

H

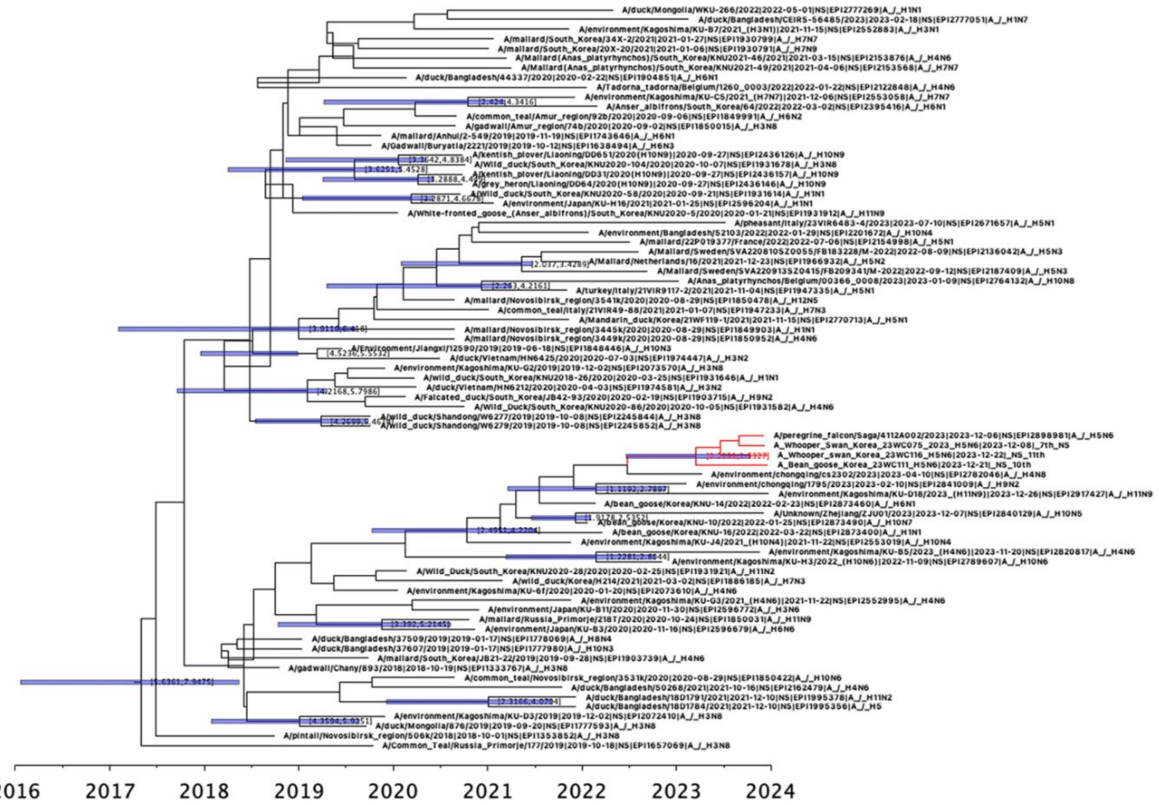

Appendix Figure 2. Bayesian phylogenetic Maximum clade credibility tree using eight genes of H5N6 HPAIV: A) PB2, B) PB1, C) PA, D) HA, E) NP, F) NA, G) MP, and H) NS. Node bars represent 95% highest posterior density of the heights at the node. H5N6 isolates from South Korea and Japan are colored in red. The time scale is shown on the horizontal axis. The scale axis indicates the branch length by year.
